# Associations between self-reported and objectively measured physical activity and overweight/obesity among adults in Kota Bharu and Penang, Malaysia

**DOI:** 10.1186/s12889-019-6971-2

**Published:** 2019-05-22

**Authors:** Yi Yi Lee, Khairil Shazmin Kamarudin, Wan Abdul Manan Wan Muda

**Affiliations:** 1 Healthy Eating and Lifestyle Practices Association (HELP), Kota Bharu, 16150 Kelantan Malaysia; 2 Center for Fundamental and Liberal Education, Universiti Malaysia Terengganu, 21030 Kuala Terengganu, Terengganu Malaysia; 3 Khazanah Research Institute, Level 25, Mercu UEM, Jalan Stesen Sentral 5, 50470 Kuala Lumpur, Malaysia

**Keywords:** Obesity, Physical activity, Body mass index, Accelerometer, Moderate-to-vigorous physical activity, Physical activity domains

## Abstract

**Background:**

For the past decades, Malaysia has seen an increased prevalence of overweight and obesity which leads to significant health threats. Physical activity is beneficial in maintaining healthy body weight. The objective of this study was to measure physical activity of adults in Malaysia using objective measurement (accelerometer) and self-reported methods, as well as to determine their associations with (body mass index (BMI) and waist circumference (WC) measurements.

**Methods:**

Four-hundred and ninety Malaysian adults (*n* = 490) aged 20 to 65 years old participated in this cross-sectional study. Their body weight, height, and WC measurements were measured according to standard procedures. Physical activity was assessed objectively with accelerometers for five to seven consecutive days. The International Physical Activity Questionnaire (IPAQ) was used to estimate the amount of time spent on various domains of physical activity. Mixed models were used to determine the associations between physical activity variables and both BMI and WC.

**Results:**

The mean value of objectively measured moderate-to-vigorous physical activity (MVPA) was 13.5 min per day, in which male participants recorded a significantly higher amount of time compared to females. On the other hand, the mean self-reported total physical activity was 380 min per week; male participants reported a significantly higher amount of time on physical activity in the occupation/work and leisure/recreation domains while female participants spent significantly more time in the domestic/household chores domain. We also observed that the mean values of objectively measured total MVPA, self-reported time spent on walking for leisure/recreation, and total time amount of time spent on MVPA for leisure/recreation were significantly higher among participants with BMI of less than 25 kg/m^2^. The final statistical model yielded a significant negative association between objectively measured total MVPA and BMI, but not with WC measurement. No significant association was reported between self-reported total physical activity with BMI and WC measurement.

**Conclusions:**

Objectively measured MVPA was inversely associated with BMI, but not WC measurement. No significant association was observed between self-reported total physical activity and physical activity time measures across domains with both BMI and WC measurement.

## Background

Obesity has tripled worldwide since 1975, reaching epidemic proportions in both developing and developed countries; as of 2018, 13% of adults are obese and 39% overweight [1]. The Global Burden of Disease Study [2] reported a prevalence of overweight and obesity in Southeast Asia of 22.1% among men and 28.3% among women. The rates were highest in Malaysia at 48.3 and 48.6% for men and women, respectively [2]. The 2015 Malaysian National Health and Morbidity Survey (NHMS) reported similar numbers, estimating the national prevalence of overweight and obesity in adults at 30.0 and 17.7%, respectively, for a total of 47.7% [3].

Two significant contributors to the development of obesity and many non-communicable diseases include poor dietary quality and insufficient physical activity [4, 5]. Physical inactivity is one of the modifiable behavioural risk factors which increases the risk of obesity and non-communicable diseases [6] and makes up 1–3% of national health care costs, excluding costs associated with mental health and musculoskeletal conditions [7]. On the contrary, regular physical activity of moderate- and vigorous-intensity prevents excessive weight gain by increasing lean body mass and resting metabolic rate [8]. It also contributes to a reduced risk of mortality, non-communicable diseases, dementia, depression, and promotes a better quality of life [8, 9]. Recent findings from Malaysia’s NHMS in 2015 showed that the national prevalence of adults who were physically active was 66.5%, and among them, 41.1% were “minimally active,” and only 25.4% were “highly active” [3].

Defined as a series of behaviours involving bodily movements produced by skeletal muscles that requires energy expenditure [10], physical activity includes a wide range of activities, such as walking, exercising, swimming, dancing, working, playing, carrying out domestic and household chores, travelling from place to place (transportation), and engaging in recreational activities [11]. The World Health Organization (WHO) recommends at least 150 min of moderate-intensity physical activity or 75 min of vigorous-intensity physical activity throughout the week, or more for its health benefits [7]. An updated version of the “2018 Physical Activity Guidelines for Americans” by the 2018 Physical Activity Guidelines Advisory Committee recommends that adults should perform at least 150 min to 300 min a week of moderate-intensity, 75 to 150 min a week of vigorous-intensity aerobic physical activity, or an equivalent combination of both moderate- and vigorous-intensity aerobic activity, and muscle-strengthening activities on 2 or more days a week [9].

The development of methods to measure physical activity is an important area of methodological development in the attempt to study and understand the correlates of physical activity behaviour [11]. Physical activity is commonly assessed using questionnaires, direct observation, physical activity diary, direct measurement of physical activity, or a combination of several methods [12]. Research in physical activity relies heavily on the questionnaire methods, where they predominantly describe structured movement performed during exercise, sport, and work [13, 14]. However, recent advancement in objective measurements has enabled researchers to measure the entire range of activities, including sedentary, light, moderate, and vigorous in free-living subjects for several days, and obtain the cumulative time spent each day on activities of all intensities [15]. Physical activity is usually described using four parameters, namely (i) type (referring to the main physiological systems that are activated during the activity, such as aerobic or anaerobic); (ii) frequency (the number of times performed over a period); (iii) duration (representing the length of the activity and usually quantified in minutes); and (iv) intensity (relating to the degree to overloading the activity imposes on physiological systems compared to resting states and often described as mild, moderate, or vigorous) [16]. Physical activity can be categorized into several domains, namely occupational (work or job related), transportation (walking and cycling); domestic (household chores, yard-work, child-care); and leisure-time (recreational time for physical activity, exercise, sports, and hobbies) physical activity [17]. Accurate assessment of physical activity is extremely critical in the discussions of its benefit to health [11].

In Malaysia, previous studies had shown cross-sectional and longitudinal associations between objectively measured and self-reported physical activity with different measures of overweight and obesity [16–22]. Existing physical activity studies that are specific to the Malaysian population often depended on self-reported measures [21, 23], while accelerometer studies are still limited and had small sample sizes [24–26]. Physical activity assessment among free-living Malaysian adults with both objective and self-reported measures according to domains and their associations with overweight/obesity has yet to be explored extensively [22, 27].

The purpose of this study was to measure physical activity using self-reported and objective measures (accelerometer) in a sample of Malaysian adults and to also determine the association of these physical activity variables with body mass index (BMI) and waist circumference (WC) measurements. Physical activity was analysed stratified by sex and BMI (< 25 kg/m^2^ and ≥ 25 kg/m^2^).

## Methods

### Study design

This cross-sectional study was conducted in two major cities in Malaysia, namely Kota Bharu and Penang, and was a part of a more extensive study on neighbourhood environment, physical activity, and nutritional status among Malaysian adults. Four hundred and ninety (*n* = 490) Malaysian adults aged between 20 to 65 years old sampled via a multistage sampling method participated in this study. They were sampled from neighbourhoods (administrative units) that were initially stratified according to the walkability indices of their neighbourhoods [28]. Adults who were considered eligible to participate were those who agreed to wear the accelerometer, sign the informed consent form, and can walk. Individuals living in group living establishments (e.g., hostels, nursing homes) were excluded from this study. The study was approved by the Universiti Sains Malaysia Research Ethics Committee (Human) (FWA Reg. No: 00007718; IRB Reg. No: 00004494) (Reference Number: (USMKK/PPP/JePeM [246.3(6)(1)])). The response rate was 65.3%.

### Instruments

#### Sociodemographic information

The participants responded to questions on their sociodemographic background, including age, sex, ethnicity, level of education, household size, marital status, employment status, and total household monthly income.

#### Anthropometric measurements

The standing height was measured using a SECA bodymeter (Model seca 203, seca gmbh & co. kg., Hamburg, Germany) according to standard procedures to the nearest 0.1 cm. Body weight was measured using a SECA digital weighing scale (Model seca clara 803, seca gmbh & co. kg., Hamburg, Germany) with an accuracy of 0.1 kg while adhering to the standard procedures. The WHO BMI classification for adults [1] was used to determine the BMI status of the participants. BMI cut-offs of < 25.0 kg/m^2^ and ≥ 25.0 kg/m^2^ were used to stratify body weight status for further statistical analysis. WC was measured using a measuring tape at the point of the minimal waist according to standard procedures [29].

#### Objectively measured physical activity

Actigraph GT3X+ physical activity monitors or accelerometers (Actigraph, Florida, USA) were used to obtain objective measurements of physical activity. Actigraph devices are widely used and have been extensively validated [30, 31]. For this study, each participant was asked to wear an accelerometer on their waists during waking hours for at least twelve hours per day for seven consecutive days, during which movements were recorded in 60-s time interval, termed “epoch.” The accelerometer data were screened and scored using the ActiLife Version 6.13.2 software (Actigraph, Florida, USA) and MeterPlus 4.3 software (Santech Inc., California, USA). A valid accelerometer hour has no more than 60 consecutive ‘zero’ value or activity counts, while a valid accelerometer wearing day must consist of at least 10 h of wearing per day. Participants who had at least 5 or more valid days (inclusive of one weekend day) recorded were accepted as successful accelerometer wearing. Participants were asked to re-wear the accelerometer if they did not achieve the minimum valid wearing days required.

For this study, we used Freedson’s cut-off point of 1952 counts per minute (cpm) for moderate intensity physical activity to derive the outcome variable of mean minutes of moderate-to-vigorous physical activity (MVPA) per valid day [32]. These activity count cut-off points were then applied to the accelerometer data and total minutes at or above the MVPA threshold were obtained from each participant (inclusive of moderate, vigorous and very vigorous physical activity). Total minutes of MVPA achieved in a successful wearing were then divided by the number of valid accelerometer-wearing days to obtain the average total MVPA minutes per day. These methods were consistent with the recommendations and standard practices made in previous studies [33, 34].

#### Self-reported physical activity: international physical activity questionnaire (IPAQ) – long form

The IPAQ – Long-Form is an extensively validated and commonly used measurement tool to estimate physical activity and sedentary behaviour of adults aged 15 to 69 years [35, 36]. It consists of a set of 27 questions which reflect on the previous seven days’ activities according to life domains: a) occupational/job-related physical activity; b) transportation physical activity; c) housework, house maintenance, and caring for the family; d) recreation, sport, and leisure-time physical activity; and e) time spent sitting [35]. The questionnaire was self-administered before wearing an accelerometer to avoid changes in self-reported physical activity pattern that may arise from wearing a physical activity monitor. The participants were carefully assisted by the research staff during the completion of IPAQ to avoid confusion.

A reference period of the “last seven days” was used to assess the frequency and duration of moderate and vigorous intensity activities. According to the IPAQ definition and protocols, the physical activity reported should only involve activities that last at least 10 min at a time. [35, 36]. For analysis purposes, the frequency (number of days in the last seven days) and duration (minutes per day) of physical activity in different domains were calculated to obtain the total minutes of activity in one week. The reported minutes per week (min/week) of transport-related walking and cycling were calculated by multiplying frequency per week with duration per day. The leisure-time physical activity consisted of weekly minutes of walking for leisure and moderate and vigorous leisure-time physical activity. Walking for leisure or recreation also served as a separate variable to evaluate the time spent on walking as a leisure-time physical activity.

### Statistical analysis

Both descriptive and inferential statistical analyses were performed using the IBM SPSS Statistics for Windows, Version 25.0 (Armonk, NY, USA). Descriptive statistics are presented as the mean, standard deviation, and 95% confidence interval unless stated otherwise. The level of significance was set at *p* < 0.05.

The MIXED model was used to determine associations between physical activity variables and the obesity indicators. In a MIXED model, responses from a participant are thought to be the sum (linear) of the fixed or random effects. For example, a fixed effect affects the population mean (such as a medical treatment), while a random effect is an effect associated with a sampling procedure (subject effect). The random effect in this study is the subject effect, as the participants were sampled for multiple neighbourhoods in two different cities in the country. MIXED models also allow the modelling of data with skewed distributions, which are typical of physical activity data, while accounting for clustering effects arising from a multistage sampling strategy [37]. The covariates included in the MIXED models’ analysis were age, sex, marital status, education level, employment status, city or study site, socioeconomic status (SES), and the average of accelerometer wearing hours per day. These are common sociodemographic factors that might affect the outcome of the study [38, 39].

## Results

Table 1 showed the background information and characteristics of the participants. Among them, 64.7% (*n* = 317) were women; 61.8% (*n* = 303) were married or living with a partner; 50.8% (*n* = 249) were employed or had unpaid work outside the home; and 33.9% (*n* = 166) had tertiary education (diploma and above). In terms of ethnicity, there were 71.4% (*n* = 350) Malays and 25.7% (*n* = 126) Chinese, while the remaining participants were Indians and Sikhs. The participants represented various ethnicities of Malaysians. The mean age of the participants was 40.0 (14.3) years. Less than 50% of participants had a total household income of Malaysian Ringgit 3000.00 or higher. Using the WHO BMI classification, 52.8% of the participants were either overweight or obese with BMI above 25 kg/m^2^.

**Table 1:** General characteristics, obesity, and physical activity status of participants

Characteristics	*n*	%	Mean (SD)
Age (years)			40.0 (14.3)
Household size			4.9 (2.3)
BMI (kg/m^2^)			25.9 (6.0)
WC measurements (cm)			82.6 (14.2)
Sex			
Female	317	64.7	
Male	173	35.3	
City			
Penang	196	40.0	
Kota Bharu	294	60.0	
Marital status			
Married	303	61.8	
Not married	187	38.2	
Employment status			
Does not have a job or an unpaid job outside the home	241	49.2	
Has a job or unpaid job outside the home	249	50.8	
Education level			
Did not complete upper secondary school education (or equivalent)	123	25.1	
Completed upper secondary school education (or equivalent)	201	41.0	
Tertiary education or equivalent (Diploma and above)	166	33.9	
Total household monthly income (Self-reported)			
Below MYR 3000.00^1^	345	70.3	
Above MYR 3000.00	145	29.6	
Obesity status (BMI in kg/m^2^)			
BMI < 25.0	231	47.2	
BMI 25.0–29.9	151	30.8	
BMI ≥ 30.0	108	22.0	
Achieved 30 min MVPA per day^c^ (accelerometer^a^)			
Yes	55	11.2	
No	432	88.2	
Missing accelerometer data	3	0.6	
Achieved 20 min MVPA per day (accelerometer^a^)			
Yes	111	22.7	
No	376	76.7	
Missing accelerometer data	3	0.6	
Self-reported total physical activity (IPAQ – Long Form^b^)			
< 150 min per week	176	35.9	
> 150 min per week	314	64.1	
< 300 min per week	278	56.7	
> 300 min per week	212	43.3	

*n* number, *SD* standard deviation, *BMI* body mass index, *WC* waist circumference, *MVPA* moderate-to-vigorous physical activity, *IPAQ* International Physical Activity Questionnaire

^1^1.00 Malaysian Ringgit (MYR) is equivalent to 0.25 United States Dollar (USD); ^a^measured in minutes/day; ^b^ measured in minutes/week; ^c^WHO recommendation for MVPA

Based on accelerometer-measured MVPA, only 11.2% of the participants achieved 30 min of MVPA per day, as per recommendations of 30 min of moderate physical activity on most days of the week by the WHO [7]. When categorized according to the 20 min of MVPA per day cut-off point, the proportion of participants achieving the cut-off point was only 22.7%. On the other hand, based on the recent weekly physical activity recommendations [9], results from the IPAQ showed that 64.1% of the participants achieved more than 150 min of self-reported physical activity per week. If categorized according to the 300 min per week cut-off, only 43.4% of the participants reached this cut-off point.

The objectively measured and self-reported physical activity variables were displayed in Table 2. The mean minutes of total MVPA per day recorded by accelerometers were 13.5 (14.0) min/day. For self-reported physical activity measures, the mean minutes of total physical activity were 380 min/week. Meanwhile, the mean time spent on active transport (walking and biking) was 201.9 (368.4) min/week while walking for leisure and recreation was 91.7 (208.0) min/week. The large standard deviation values in the self-reported physical activity variables were caused by the ‘zero’ reported minutes (no physical activity) and occasionally over-reported minutes of physical activity in the IPAQ. Thus, median values were also reported in Table 2.

**Table 2:** Differences in obesity and physical activity indicators grouped by sex

Outcome	Total			Sex		Mean Difference	95% CI for Mean Difference
				Male	Female		
	Mean (SD)	Median	IQR	Mean (SD)	Mean (SD)		
BMI (kg/m^2^)*	25.9 (6.0)	25.3	7.6	25.0 (5.5)	26.4 (6.1)	−1.38	−2.48, −.28
Waist circumference measurement (cm)*	82.6 (14.2)	82.5	19.9	85.5 (15.3)	(81.1) 13.4	4.38	1.74, 7.03
Objective measurement of physical activity (accelerometer)^a^							
Total MVPA per day*	13.5 (14.0)	8.0	14.5	17.4 (14.5)	(11.3) 13.3	6.13	3.56, 8.71
Self-reported physical activity^b^							
Total self-reported physical activity	379.9 (466.0)	243.5	383.2	425.4 (540.8)	355.1 (418.5)	70.33	−16.09, 156.75
Total physical activity at work [including walking]*	103.0 (174.2)	0.0	139.3	129.1 (203.9)	88.7 (154.1)	40.39	8.20, 72.58
MVPA at work [excluding walking]	65.5 (124.5)	0.0	72.1	85.4 (149.5)	51.6 (106.9)	33.85	10.89, 56.80
Biking as transport	12.9 (83.3)	0.0	0.0	17.0 (90.9)	10.5 (78.8)	6.44	−9.05, 21.9
Walking as transport	189.8 (353.1)	70.0	210.0	222.0 (397.3)	171.6 (325.2)	50.41	−15.07, 115.90
Total active transport [walking and biking]	201.9 (368.4)	70.0	210.0	239.0 (404.4)	181.6 (368.3)	57.47	−10.83, 125.76
Total physical activity during housework, house maintenance and caring for family*	66.8 (88.1)	34.3	84.1	45.2 (66.1)	78.5 (96.2)	−33.28	−49.40, −17.16
Walking for leisure and recreation*	91.7 (208.0)	0.0	90.0	130.4 (299.1)	70.5 (130.0)	59.98	21.64, 98.30
MVPA for leisure and recreation*	8.1 (23.8)	0.0	0.0	11.9 (26.4)	6.2 (22.1)	5.75	1.35, 10.167

*n* number, *SD* standard deviation, *IQR* interquartile range, *MVPA* moderate-to-vigorous physical activity

^a^ measured in minutes/day; ^b^ measured in minutes/week; *p < .05

The results of *t*-test for BMI, WC measurements, accelerometer-measured physical activity data, and self-reported IPAQ – Long Form physical activity data were grouped according to sex (Table 2). Female participants recorded significantly higher BMI, whereas male participants had significantly larger waist circumference measurements. We observed that male participants were significantly more active physically, recording a mean total MVPA of 17.4 min/day compared to female participants who recorded a mean total MVPA of 11.3 min/day. For self-reported physical activity, no significant difference in total physical activity was observed among male and female participants. Male participants reported significantly longer duration (minutes) of total physical activity at work (inclusive of walking at work) and longer duration of walking and MVPA for leisure/recreation. No significant difference was found in the total active transport time. Meanwhile, female participants reported a significantly longer duration of physical activity in the housework, house maintenance, and caring for the family domain.

Subsequently, the participants were categorized into two groups, one with BMI less than 25 kg/m^2^ and another with BMI equals to or more than 25 kg/m^2^, as displayed in Table 3. The mean values of objectively measured total MVPA, time spent walking for leisure, and time spent on MVPA for leisure/recreation were significantly lower among participants with BMI ≥ 25 kg/m^2^. The mean value of time spent on doing housework/domestic chores domain was found to be significantly higher among participants with BMI ≥ 25 kg/m^2^.

**Table 3:** Differences in waist circumference and physical activity indicators grouped by body weight status (BMI < 25 kg/m^2^ vs. BMI ≥ 25 kg/m^2^)

Outcome	Group						Mean Difference	95% CI for Mean Difference
	BMI < 25 kg/m^2^			BMI ≥ 25 kg/m^2^				
	M	SD	*n*	M	SD	*n*		
Waist circumference measurement (cm)**	72.9	10.2	231	91.1	11.6	259	−18.23	−20.20, −16.23
Objective measurement of physical activity (accelerometer)^a^								
Total MVPA per day**	17.5	16.9	229	9.8	9.6	258	7.68	5.26, 10.11
Self-reported physical activity^b^								
Total self-reported physical activity	378.1	524.7	231	381.5	407.8	259	−3.33	−82.29, 79.62
Total physical activity at work [including walking]	93.8	181.5	231	111.3	167.4	259	−17.46	15.76, −48.43
MVPA at work [excluding walking]	54.9	122.3	231	71.2	126.2	259	−16.27	−38.40, 5.84
Biking as transport	12.6	82.2	230	13.1	85.1	258	4.55	−10.31, 19.40
Walking as transport	155.4	290.8	230	240.3	424.9	259	31.15	−31.67, 93.97
Total active transport [walking and biking]	220.3	406.6	231	185.5	330.5	259	34.78	−30.71, 100.27
Total physical activity during housework, house maintenance and caring for family**	52.9	82.7	231	79.4	91.0	259	−26.54	−42.06, −11.03
Walking for leisure and recreation	99.1	240.3	230	85.0	174.6	259	14.03	−23.02, 51.08
MVPA for leisure and recreation*	11.2	29.3	231	5.3	17.2	259	5.90	1.68, 10.11

*n* number, *SD* standard deviation, *IQR* interquartile range, *MVPA* moderate-to-vigorous physical activity

^a^measured in minutes/day; ^b^measured in minutes/week; *p < .05, **p < .01

Mixed models were used to determine the association between the objectively measured and self-reported physical activity variables with BMI (Table 4) and WC measurements (Table 5). Objectively measured MVPA was negatively associated with BMI (*p* < 0.05). We did not find any significant association between physical activity variables with WC measurement.

**Table 4:** Associations between objective total MVPA and self-reported PA with BMI

Variables	Estimate *b*	*t*	95% CI	*p*
Objective measurement of physical activity				
Total MVPA per day	−.045	−2.161	−.087, −.004	< .050
Self-reported physical activity				
Total self-reported physical activity	−.0001	.629	−.0012, .001	.856
Total physical activity at work [including walking]	.002	1.166	−.001, .006	.244
MVPA at work [excluding walking]	.004	1.858	−.0002, .009	.064
Biking as transport	−.003	−1.074	−.009, .002	.283
Walking as transport	−.0005	−.675	−0.001, 0.001	.050
Total active transport [walking and biking]	−.0006	−.845	−.002, .0007	.399
Total physical activity during housework, house maintenance and caring for family	.003	1.037	−.002, .009	.300
Walking for leisure and recreation	−.0005	−.448	−.002, .002	.654
MVPA for leisure and recreation	−.013	−1.184	0.034, .008	.237

*MVPA* moderate-to-vigorous physical activity

Note. The dependent variable for the model is BMI. Age, sex, marital status, education, employment status, city (site for data collection), socioeconomic status (SES), and the average of valid accelerometer wearing hours per day were included as covariates in all models

**Table 5:** Associations between objective total MVPA and self-reported PA with WC measurements

Variables	Estimate *b*	*t*	95% CI	*p*
Objective measurement of physical activity				
Total MVPA per day	−.061	− 1.27	−.15, .033	.204
Self-reported physical activity				
Total self-reported physical activity	−.0003	.-.251	−.002, 0.002	.802
Total physical activity at work [including walking]	.0007	.179	−.007, .008	.858
MVPA at work [excluding walking]	.004	.886	−.006, .015	.376
Biking as transport	−.004	−.499	−.017, .010	.618
Walking as transport	−.0005	−.339	−.003, .003	.735
Total active transport [walking and biking]	−.0006	−.397	−.003, .002	.692
Total physical activity during housework, house maintenance and caring for family	0.002	.406	0.108, .016	.685
Walking for leisure and recreation	−.0002	−.091	−.005, .005	.928
MVPA for leisure and recreation	−.028	−1.144	−.076, 0.020	.253

*MVPA* moderate-to-vigorous physical activity

Note. The dependent variable for the model is the WC measurement. Age, sex, marital status, education, employment status, city (site for data collection), socioeconomic status (SES), and the average of valid accelerometer wearing hours per day were included as covariates in all models

## Discussion

Adults on average spend a large proportion of their time in sedentary behaviours and light-intensity activities during their waking hours – a typical 24-h cycle usually comprised of 7.5 h of sleeping, 9.4 h of sedentary behaviours, 6.5 h of light-intensity activities, and close to 43 min of moderate or vigorous physical activity [40]. In the present study, the objectively measured mean total MVPA among the participants was 13.5 min/day, while self-reported mean physical activity was 380 min/week. Based on the analysis, we observed that only a small proportion of participants in this study achieved the physical activity recommendations established by the WHO as the majority were not adequately active to experience the health benefits of physical activity. The mean total MVPA time of the participants (13.5 mins/day) was very low compared to a handful of other cities in a multi-country study which adopted similar measurement methods using accelerometers, with the lowest mean MVPA time reported in the city of Baltimore in Maryland, USA (29.2 mins per day) and the highest in Wellington, New Zealand (50.1 mins per day) [34].

Analysis of the self-reported physical activity data indicated that 64.1% of the participants were active and achieved more than 150 min of activity per week. These results are consistent with studies conducted among other sub-populations in Malaysia. For example, in a sample of overweight and obese women in Malaysia, 61.1% of them were categorised as physically active when the assessment was conducted using self-reported measures, but only 8.4% were categorised as being sufficiently active via objective measurement method using pedometers [22]. Soon and colleagues used the Lifecorder e-Step accelerometer to measure the total number of steps per day and the total energy expenditure in a sample of office workers in a public university in Malaysia [24]. Consistent with our findings, only a small percentage of the adult office workers (8.5%) were categorized as “active” or “highly active” based on the number of steps achieved per day.

Additionally, Soon and colleagues also reported that the total daily energy expenditure recorded (a result of physical activity) was positively correlated with BMI and WC in their study, while increased steps achieved per day was associated with a decrease in BMI [24]. Another accelerometer study on Malaysian adults found significant associations between physical activity levels and BMI and WC [25]. A survey of employees working in government agencies in the country showed that increasing BMI was linked to lower levels of physical activity [26]. However, it is essential to note that these studies [22, 24–26] measured only the steps and physical activity levels of their participants, but not the amount of time spent on MVPA per day.

Sex differences in physical activity and anthropometric indicators were observed clearly in the current study. Male participants spent significantly more time in all physical activity domains except for the transport-related domain (no difference was observed between males and females) and the housework/domestic chores domain (where females spent significantly more time). These findings are supported by evidence from a systematic review which summarized that participation of physical activity was consistently higher in men compared to women in previous studies [41]. Furthermore, an analysis on adults’ physical activity pattern across different life domains in two regions in the United States (Baltimore and Seattle) indicated that the total physical activity at work and objective-measured MVPA were significantly higher among males [42]. The nationwide 2002–2003 Malaysian Adult Nutrition Survey (MANS), NHMS III, as well as two local studies using self-reported measures of physical activity have also shown that men were more active than women in the country [43–46]. Furthermore, results from a local study indicated that homemakers were more likely to engage in moderate-intensity physical activity in the domestic or household domain rather than to the leisure-time domain due to family obligations and lack of time [47]. The trend of lower physical activity levels among the females is worrying as obesity is also more prevalent among the females in the country [3].

For self-reported physical activity measures, we found that time spent on leisure/recreation physical activity domain was lower than job-related physical activity and walking for transport and leisure domains. This trend was also observed in a more extensive, nationally representative study in the country which showed that only a small percentage of Malaysians were involved in leisure-time physical activity, and travelling by car or motorcycle is very common and preferred which resulted in the high prevalence of physical inactivity among the respondents of the survey [48]. The usage of motorised vehicles reduces walking or biking as a form of active transportation which contributes substantially to overall physical activity levels. The low participation of Malaysian adults in leisure-time physical activity is a troubling phenomenon. Since it is widely recognised that increased participation in recreational and leisure-time physical activity could compensate for lower energy expenditure within normal daily living [49], this positive behaviour or lifestyle practice should be encouraged from a young age. For instance, in Japan, participation in leisure-time physical activity in youth predicted regular leisure-time physical activity participation in working adults [50].

On the other hand, differences in BMI status (having healthy body weight and being overweight or obese) on physical activity indicators could also be observed in the present study. Time spent on self-reported physical activity for leisure and recreation as well as objectively measured physical activity were significantly higher among participants who were not overweight or obese. This observation is consistent with a previous study to determine BMI and physical activity relationship in Sweden [51]. The subjects were stratified into severely obese and non-obese groups, and physical activity was measured with accelerometers for seven consecutive days [51]. Time spent on moderate and vigorous intensity physical activity and the total volume of physical activity were significantly higher in the non-obese group compared to the obese group [51].

In the present study, after adjusting for covariates, the objectively measured total MVPA was inversely associated with BMI, but not WC measurement. No association was observed in the self-reported measures with both BMI and WC. A study on Malay adults indicated that increased self-reported physical activity was inversely associated with BMI and was protective against central obesity [45]. Wareham and colleagues (2005) reviewed several studies on physical activity and weight gain in adults and found that most of the studies reported statistically significant inverse associations between the self-reported measures of physical activity and weight gain [52]. On the other hand, results from The Korea National Health and Nutrition Examination Survey found more significant associations between physical activity and obesity defined by waist-to-hip ratios than the BMI among both Korean men and women, but no significant associations were found between physical activity and obesity by BMI in women [53].

A negative relationship was also observed in accelerometer-measured MVPA with BMI and waist circumference among a sample of Chilean adults, while no relationship was detected in self-reported IPAQ MVPA with BMI and waist circumference measurements [17]. Data from the National Health and Nutrition Examination Survey (NHANES) 2003–2006 physical activity data showed significant associations between objectively measured physical activity with measures of overweight and obesity (inclusive of BMI and WC) [19]. Only vigorous-intensity, but not the moderate-intensity self-reported physical activity was associated with the same overweight and obesity measures [19]. Similar to our study, Wanner et al. [19] observed consistent associations between objectively measured physical activity and overweight/obesity measures, whereas self-reported physical activity associations were found for vigorous but not moderate intensity physical activity. A possible explanation by Wanner et al. [19] which would also apply to the present study is that the intensity of physical activity was overestimated, and self-reported moderate-intensity PA corresponds better with accelerometer-measured light-intensity PA. Self-reported measures may be more susceptible to misclassification, especially regarding moderate-intensity PA and household tasks.

We did not find any significant association between objectively measured total MVPA per day with WC measurements in this study. We also did not detect any association between any of the self-reported physical activity in various domains with WC measurements. It is possible that the habitual and prolonged inadequacy of physical activity among most of the participants had already created an accumulation of fat in the abdominal areas before the study was conducted. A longitudinal study among middle-aged women reported that changes in sports or exercise had less influence on WC and implied that physical activity may have more impact or contributed more to prevent increases in the amount of fat rather than redistribution of fat [54].

The understanding of the differences in physical activity measurements is vital to enable comparisons between studies and for better understanding of the complexity of obesity and physical activity, as demonstrated by the findings in this current study. The tendency to overreport self-reported behaviours may weaken the associations between variables. Under-reporting and over-reporting of behaviours may also result in biased conclusions made on the correlates of physical activity behaviours [55]. However, as proven by larger-scale or cohort studies, it cannot be denied that self-reported physical activity method will remain as and continue to be an essential tool to obtain information on the volume of physical activity and identify the health consequences of physical inactivity. Even though the IPAQ were said to overestimate physical activity, it is still the most appropriate measure for this study because it provided estimates for all the four domains of physical activity [56].

As shown in the present study, objectively-measured MVPA and self-reported time spent in various physical activity domains differ significantly among males and females and participants with different body weight status. Moreover, a better understanding of physical activity and its correlates is beneficial so that public health and clinical interventions can target stronger mediators of their behaviour. The effort to increase physical activity participation will require combined strategies to involve all the individual, social, cultural, environmental, and policy determinants of physical inactivity. Other than the neighbourhood environment walkability attributes, this study did not investigate other possible barriers of physical activity, which is neither the objective of this paper nor discussed in this paper. Nevertheless, other studies conducted in the country previously indicated that family obligations; lack of time; financial constraints; health problems; lack of facilities; discouragement from spouse, family, and friends; bad weather; as well as the lack of motivation and interest are the most common barriers to physical activity participation among Malaysians [57–59].

One of the strengths of this study was the use of validated and standardized measures of both objective and self-reported measurements of physical activity that have been practiced in international studies. This has allowed an extensive and detailed assessment of the frequency, duration, and intensity of physical activity, and provided information about the mode of physical activity performed. Besides, anthropometric indicators were also measured by trained personnel using standardized methods. One of the limitations of the study includes the difficulty for participants to comply with accelerometer wearing due to the long period of physical activity monitoring needed. It was also a challenge to recruit male participants and those who are staying in residential areas with tight security.

Since this study is cross-sectional, we could not infer cause and effect. We could not determine if physical activity leads to lower BMI or if those with lower BMI were involved in more physical activity. The generalisability of the sample against the Malaysian population might be limited due to the study sample size and the recruitment approach. Nevertheless, this dataset provides rich and reliable information on objectively measured physical activity as well as self-reported physical activity across life domains among adults in Malaysia. Future studies could be applied to more diverse populations including, for instance, children, adolescents, elderly or other subgroups of individuals, although different methods of physical activity monitoring and questionnaires should be used.

## Conclusions

In conclusion, we found a significant association between objectively-measured MVPA and BMI, but not WC measurement. No significant association was found between self-reported physical activity with both BMI and WC measurement. A large proportion of the participants were also not physically active to experience the benefits of physical activity. With the alarmingly high national prevalence of overweight (30.0%) and obesity (17.7%) in the country [3] as well as the existing evidence that physical activity has a crucial role to play in combatting the increasing waistlines among Malaysians, physical activity should be further promoted and encouraged. Understanding of how adults arrange their activity pattern across multiple life domains would enable strategies, interventions, and policies to be effectively formulated for the diverse population.

## Abbreviations

BMI: Body mass index
CI: Confidence interval
IPAQ: International physical activity questionnaire
MANS: Malaysian adults nutrition survey
MVPA: Moderate-to-vigorous physical activity
NHMS: National health and morbidity survey
SD: Standard deviation
WC: Waist circumference
WHO: World Health Organization

## References

[1] World Health Organization. Obesity and Overweight. http://www.who.int/news-room/fact-sheets/detail/obesity-and-overweight. Accessed 16 Feb 2018.

[2] Ng M, Fleming T, Robinson M, Thomson B, Graetz N, Margono C, Mullany EC (2014). Global, regional, and National Prevalence of overweight and obesity in children and adults during 1980–2013: a systematic analysis for the global burden of disease study 2013. Lancet.

[3] Institute for Public Health, Ministry of Health Malaysia. National health and morbidity survey 2015 (NHMS 2015). Vol. II: Non-Communicable Diseases, Risk Factors & Other Health Problems. http://iku.moh.gov.my/images/IKU/Document/REPORT/nhmsreport2015vol2.pdf Accessed 20 May 2016.

[4] Booth FW, Roberts CK, Laye MJ. Lack of exercise ss a major cause of chronic diseases. In R. Terjung (Ed.), Comprehensive physiology. Hoboken, NJ, USA: John Wiley & Sons, Inc.; 2012. Available from http://doi.wiley.com/10.1002/cphy.c110025. Accessed 10 May 2016. 10.1002/cphy.c110025 PMC4241367 23798298

[5] Lachat C, Otchere S, Roberfroid D, Abdulai A, Seret FMA, Milesevic J (2013). Diet and physical activity for the prevention of noncommunicable diseases in low- and middle-income countries: a systematic policy review. PLoS Med.

[6] World Health Organization. Non-communicable diseases. http://www.who.int/news-room/fact-sheets/detail/noncommunicable-diseases. Accessed 27 August 2018.

[7] World Health Organization. Global Recommendations on Physical Activity for Health. http://www.who.int/dietphysicalactivity/physical-activity-recommendations-18-64years.pdf?ua=1. Accessed 26 May 2016.

[8] Miles L (2007). Physical activity and health. Nutr Bull.

[9] U.S. Department of Health and Human Services. 2018 Physical activity guidelines advisory committee. 2018 physical activity guidelines advisory committee scientific report. https://health.gov/paguidelines/second-edition/report/pdf/pag_advisory_committee_report.pdf. Accessed 1 Dec 2018.

[10] Caspersen CJ, Powell KE, Christenson GM (1985). Physical activity, exercise, and physical fitness: definitions and distinctions for health-related research. Public Health Rep.

[11] Panter J, Griffin S, Ogilvie D (2012). Correlates of reported and recorded time spent in physical activity in working adults: results from the commuting and health in Cambridge study. PLoS One.

[12] Hills AP, Mokhtar N, Byrne NM. Assessment of physical activity and energy expenditure: an overview of objective measures. Front Nutr. 2014;1. 10.3389/fnut.2014.00005. 10.3389/fnut.2014.00005 PMC4428382 25988109

[13] Janz KF (2006). Physical activity in epidemiology: moving from questionnaire to objective measurement. Brit J Sport Med.

[14] Castillo-Retamal M, Hinckson EA (2011). Measuring physical activity and sedentary behaviour at work: a review. Work..

[15] Godfrey A, Conway R, Meagher D, ÓLaighin G (2008). Direct measurement of human movement by accelerometry. Med Eng Phys.

[16] Tudor-Locke C, Brashear MM, Johnson WD, Katzmarzyk PT (2010). Accelerometer profiles of physical activity and inactivity in normal weight, overweight, and obese U.S. men and women. Int J Behav Nutr Phys Act.

[17] Wanner M, Martin BW, Autenrieth CS, Schaffner E, Meier F, Brombach C, Stolz D, Bauman A, Rochat T, Schindler C, Kriemier S, Probst-Hensch N (2016). Associations between domains of physical activity, sitting time, and different measures of overweight and obesity. Prev Med Rep.

[18] Celis-Morales CA, Perez-Bravo F, Ibañez L, Salas C, Bailey MES, Gill JMR (2012). Objective vs. self-reported physical activity and sedentary time: effects of measurement method on relationships with risk biomarkers. PLoS One.

[19] Wanner M, Richard A, Martin B, Faeh D, Rohrmann S (2017). Associations between self-reported and objectively measured physical activity, sedentary behavior and overweight/obesity in NHANES 2003-2006. Int J Obes.

[20] Innerd P, Harrison R, Coulson M (2018). Using open source accelerometer analysis to assess physical activity and sedentary behaviour in overweight and obese adults. BMC Public Health.

[21] Chan YY, Lim KK, Lim KH, Teh CH, Kee CC (2017). Physical activity and overweight/obesity among Malaysian adults: findings from the 2015 National Health and morbidity survey (NHMS). BMC Public Health.

[22] Mohamad Hasnan A, Ruhaya S, Noor Safiza MN, Azli B, Wan Shakira RH, Azahadi O, Ahmad Taufik J, Mahenderan A, Wan Abdul Manan WM, Tahir A (2018). Comparison between self-reported physical activity (IPAQ-SF) and pedometer among overweight and obese women in the MyBFF@home study. BMC Womens Health.

[23] Chan YY, Lim KK, The CH, Kim KH, Hamizatul Akmal AH (2014). Prevalence and factors associated with physical inactivity among Malaysian adults. Southeast Asian J Trop Med Public Health.

[24] Soon HK, Saad HA, Taib MNM, Rahman HA, Mun CY (2011). Accelerometer-determined physical activity level in adults with abdominal obesity. Int J Sport Health Sci.

[25] Hazizi AS, Aina Mardiah B, Mohd Nasir MT, Zaitun Y, Hamid Jan JM, Tabata I (2012). Accelerometer-determined physical activity level among government employees in Penang, Malaysia. Malays J Nutr.

[26] Kalmi ZN, Saad HA, Taib MNM, Yassin Z, Tabata I (2012). Objective assessment of physical activity in the workplace setting. Pakistan J Nutr.

[27] Chee HP, Hazizi AS, Barakatun Nisak MY, Mohd Nasir MT (2016). Comparison of accelerometer-based measurement with the international physical activity questionnaire (long form) in the assessment of physical activity level. Malaysian J Public Health Med.

[28] Lee YY, Narimah S, Wan Abdul Manan WM (2017). Accelerometer-measured physical activity and its relationship with body mass index (BMI) and waist-circumference (WC) measurements: a cross-sectional study on Malaysian adults in Penang and Kota Bharu. Mal J Nutr.

[29] Ross R, Berentzen T, Bradshaw AJ, Janssen I, Kahn HS, Katzmarzyk PT, Kuk JL, Seidell JC, Snijder MB, Sørensen TI, Després JP (2008). Does the relationship between waist circumference, morbidity and mortality depend on measurement protocol for waist circumference?. Obes Rev.

[30] Skotte J, Korshøj M, Kristiansen J, Hanisch C, Holtermann A (2014). Detection of physical activity types using triaxial accelerometers. J Phy Act Health.

[31] Santos-Lozano A, Santín-Medeiros F, Cardon G, Torres-Luque G, Bailón R, Bergmeir C, Ruiz JR, Lucia A, Garatachea N (2013). Actigraph GT3X: validation and determination of physical activity intensity cut points. Int J Sports Med.

[32] Freedson PS, Melanson E, Sirard J (1998). Calibration of the computer science and applications, Inc. accelerometer. Med Sci Sports Exerc.

[33] Winkler EAH, Gardiner PA, Clark BK, Matthews CE, Owen N, Healy GN (2012). Identifying sedentary time using automated estimates of accelerometer wear time. Br J Sports Med.

[34] Sallis JF, Cerin E, Conway TL, Adams MA, Frank LD, Pratt M, Owen N (2016). Physical activity in relation to urban environments in 14 cities worldwide: a cross-sectional study. Lancet.

[35] Craig CL, Marshall AL, Sjöström M, Bauman AE, Booth ML, Ainsworth BE, Pratt M, Ekelund U, Yvgve A, Sallis JF, Oja P (2003). International physical activity questionnaire: 12-country reliability and validity. Med Sci Sport Exer.

[36] Hagstromer M, Oja P, Sjostrom M (2006). The international physical activity questionnaire (IPAQ): a study of concurrent and construct validity. Public Health Nutr.

[37] Van Dyck D, Cerin E, Conway TL, De Bourdeaudhuij I, Owen N (2013). Perceived neighbourhood environmental attributes associated with adults’ leisure-time physical activity: findings from Belgium, Australia and the USA. Health & Place.

[38] Hagströmer M, Oja P, Sjöström M (2007). Physical activity and inactivity in an adult population assessed by accelerometry. Med Sci Sports Exerc.

[39] Bergman P, Grjibovski AM, Hagströmer M, Bauman A, Sjöström M (2008). Adherence to physical activity recommendations and the influence of socio-demographic correlates - a population-based cross-sectional study. BMC Public Health.

[40] Norton K, Norton L, Sadgrove D (2010). Position statement on physical activity and exercise intensity terminology. J Sci Med Sport.

[41] Rhodes RE, Mark RS, Temmel CP (2012). Adult sedentary behaviour. Am J Prev Med.

[42] Rovniak LS, Sallis JF, Saelens BE, Frank LD, Marshall SJ, Norman GJ, Conway TL, Cain KL, Hovell MF (2010). Adults’ physical activity patterns across life domains: cluster analysis with replication. Health Psychol.

[43] Poh BK, Safiah MY, Tahir A, Siti HM, Siti N, Norimah AK, Wan Manan WM, Mirnalini K, Zalilah MS, Azmi MY, Fatimah S (2010). Physical activity pattern and energy expenditure of Malaysian adults: findings from the Malaysian adult nutrition survey (MANS). Malays J Nutr.

[44] Cheah YK, Poh BK (2014). The determinants of participation in physical activity in Malaysia. Osong Public Health Res Perspect.

[45] Chu AHY, Moy FM (2014). Association between physical activity and metabolic syndrome among Malay adults in a developing country. Malaysia. J Sci Med Sport.

[46] Cai Lian T, Bonn G, Si Han Y, Chin Choo Y, Chee Piau W (2016). Physical activity and its correlates among adults in Malaysia: a cross-sectional descriptive study. PLoS One.

[47] Tan ZY, Yim HS (2010). Weight status, body image perception and physical activity of Malay housewives in Kampung Chengkau ulu, Negeri Sembilan. Int J Advancement Sci Arts.

[48] Jamil AT, Singh R, Ismail A, Omar A (2015). Non-leisure time physical activity for adult Malaysian and determinant factors. Malays J Public Health Med.

[49] Fox KR, Hillsdon M (2007). Physical activity and obesity. Obes Rev.

[50] Itoh H, Kitamura F, Hagi N, Mashiko T, Matsukawa T, Yokoyama K. Leisure-time physical activity in youth as a predictor of adult leisure physical activity among Japanese workers: a cross-sectional study. Environ Health Prev Med. 2017;37. 10.1186/s12199-017-0648-1. 10.1186/s12199-017-0648-1 PMC5664430 29165133

[51] Hemmingsson E, Ekelund U (2007). Is the association between physical activity and body mass index obesity dependent?. Int J Obesity.

[52] Wareham NJ, van Sluijs EMF, Ekelund U (2005). Physical activity and obesity prevention: a review of the current evidence. P Nutr Soc.

[53] Lee O, Lee D, Lee S, Kim YS (2016). Associations between physical activity and obesity defined by waist-to-height ratio and body mass index in the Korean population. PLoS One.

[54] Sternfeld B, Wang H, Quesenberry CP, Abrams B, Everson-Rose SA, Greendale GA, Matthews KA, Torrens JI, Sowers M (2004). Physical activity and changes in weight and waist circumference in midlife women: findings from the study of Women’s health across the nation. Am J Epidemiol.

[55] Cleland VJ, Schmidt MD, Salmon J, Dwyer T, Venn A (2011). Correlates of pedometer-measured and self-reported physical activity among young Australian adults. J Sci Med Sport.

[56] Rzewnicki R, Auweele YV, Bourdeaudhuij ID (2003). Addressing overreporting on the international physical activity questionnaire (IPAQ) telephone survey with a population sample. Public Health Nutr.

[57] Suraya I, Norimah AK, Ng LO, Wan Zurinah WN (2013). Perceived physical activity barriers related to body weight status and sociodemographic factors among Malaysian men in Klang Valley. BMC Public Health.

[58] Justine M, Azizan A, Hassan V, Salleh Z, Manaf H (2013). Barriers to participation in physical activity and exercise among middle-aged and elderly individuals. Singap Med J.

[59] Lim KC, Muhammad MY, Ahmad Tajuddin O, Mohd Salleh A, Gunathevan E, Hamdan MA, Mohd Sofian OF (2016). Examining sport and physical activity participation, motivations and barriers among young Malaysians. Asian Soc Sci.

